# Possible Role of Mother-Daughter Vocal Interactions on the Development of Species-Specific Song in Gibbons

**DOI:** 10.1371/journal.pone.0071432

**Published:** 2013-08-12

**Authors:** Hiroki Koda, Alban Lemasson, Chisako Oyakawa, Rizaldi, Joko Pamungkas, Nobuo Masataka

**Affiliations:** 1 Primate Research Institute, Kyoto University, Inuyama, Aichi, Japan; 2 Université de Rennes 1, EthoS “Ethologie Animale et Humaine” UMR6552-CNRS, Station Biologique de Paimpont, Paimpont, France; 3 Institut Universitaire de France, Paris, France; 4 Graduate School of Agricultural Science, Tohoku University, Furukawa, Miyagi, Japan; 5 Department of Biology, Andalas University, Padang, West Sumatra, Indonesia; 6 Primate Research Center, Bogor Agricultural University, Bogor, Indonesia; Institut Pluridisciplinaire Hubert Curien, France

## Abstract

Mother-infant vocal interactions play a crucial role in the development of human language. However, comparatively little is known about the maternal role during vocal development in nonhuman primates. Here, we report the first evidence of mother-daughter vocal interactions contributing to vocal development in gibbons, a singing and monogamous ape species. Gibbons are well known for their species-specific duets sung between mates, yet little is known about the role of intergenerational duets in gibbon song development. We observed singing interactions between free-ranging mothers and their sub-adult daughters prior to emigration. Daughters sang simultaneously with their mothers at different rates. First, we observed significant acoustic variation between daughters. Co-singing rates between mother and daughter were negatively correlated with the temporal precision of the song’s synchronization. In addition, songs of daughters who co-sang less with their mothers were acoustically more similar to the maternal song than any other adult female’s song. All variables have been reported to be influenced by social relationships of pairs. Therefore those correlations would be mediated by mother-daughter social relationship, which would be modifiable in daughter’s development. Here we hypothesized that daughters who co-sing less often, well-synchronize, and converge acoustically with the maternal acoustic pattern would be at a more advanced stage of social independence in sub-adult females prior to emigration. Second, we observed acoustic matching between mothers and daughters when co-singing, suggesting short-term vocal flexibility. Third, we found that mothers adjusted songs to a more stereotyped pattern when co-singing than when singing alone. This vocal adjustment was stronger for mothers with daughters who co-sang less. These results indicate the presence of socially mediated vocal flexibility in gibbon sub-adults and adults, and that mother-daughter co-singing interactions may enhance vocal development. More comparative work, notably longitudinal and experimental, is now needed to clarify maternal roles during song development.

## Introduction

Recent studies have demonstrated greater complexity and flexibility in the vocal signaling systems of nonhuman primates than was previously assumed [1]–[4]. For example, some nonhuman primates use semantic sound combinations [5]–[7], and some adult nonhuman primates modify call structures by matching the acoustic structure of their own calls to those of their affiliative partners [8]–[13]. However, the majority of early empirical data on vocal development emphasizes a disparity between humans and other primates. These previous studies revealed that immature monkeys and apes, unlike human infants, possess a fixed vocal repertoire with adult-like acoustic structures [14]–[16]. Experiments using deafening and social isolation have not provided convincing evidence for vocal learning in the production domain [16] although some authors have noted that immature nonhuman primates are particularly sensitive to adult models when learning how to use calls in an appropriate context [reviewed in 15]. Furthermore, vocal development is not entirely determined by genetic factors [17]–[19]. As older female monkeys are often the central node of the vocal network, it is possible that they may serve as potential models [20].

In humans, the mother is the primary model for language acquisition [2], [21]. In particular, maternal attachment acts to enhance the acquisition of speech sounds and further shape communication styles during human infancy [2], [21]–[24]. Vocal abilities of human infants rapidly change and mature as a result of the experience of communicative interactions with mothers or caregivers during the first two years of life [2]. At the perceptual level, adult speech inputs canalize the development of the phonological categories from 8 to 10 months old, leading to the formation of perceptual categories [25], [26]. Caregivers switch to a speech style characterized by a slower rate of speech, a higher fundamental frequency, and greater pitch modification [2], [27], known as infant-directed stereotypic speech or “motherese.” Recent experiments demonstrate that social interactions and auditory feedback using motherese facilitate vocal learning [23], [24]. However, while maternal influence on human infant language development is well established, the maternal influence on vocal development in apes and monkeys is still open to debate. The present study thus examined variation in daughters’ vocal structures, relationships between vocal variation and mother-daughters’ vocal interactions, and possible roles of mother-daughter vocal interactions on daughters’ vocal development of agile gibbons, a highly vocal ape.

Gibbons are well-known for their characteristic species-specific loud calls, or “songs,” composed of temporally organized consecutive notes often sung in concurrent pairs [28], [29]. Gibbons are, biologically, a highly divergent group with at least 14 species and four genera within the current classification [30]. Typically, their duets are characterized by the temporal coordination of stereotyped songs between pair mates [31]. In most species, males and females alternate their song within a duet, avoiding extensive overlapping [32]. Songs are sex-specific, with a very different acoustic structure in adult males and females. However, the songs of adult females, particularly the so-called “great call” section, are considerably more complex than male songs [32]. A basic function of great calls would be to strengthen the pair bond between adult female and male [33].

While gibbon vocal behaviors have garnered considerable interest, our understanding of the ontogeny of gibbon calls remains extremely limited. Newborn, juvenile, and adolescent females do not produce adult-like great calls [34], [35]. Vocalizations during juvenile and adolescent periods are immature without any salient acoustic structures of great calls. The basic acoustic structure (species-specific overall acoustic pattern) of great calls is fully acquired by approximately six years, the start of the sub-adult age class [34], [35]. Sub-adult daughters regularly involve themselves in synchronized and overlapping mother-daughter great call interactions [34], [35]. These co-singing interactions with mothers likely provide an opportunity for socially immature daughters to refine the acoustic structure of their great call. Gibbons are ideal models to examine potential maternal influences on nonhuman primate vocal development, notably due to those mother-daughter co-singing interactions which are unique in ape. However, this hypothesis has never been tested. A previous hybridization study found the development of the gibbon song to be strongly determined by genetic factors, reporting that daughters produced songs with an acoustic intermediate pattern between both parents’ species-specific songs [34]. So far, the idea that mothers could contribute to song development in gibbons has been neglected probably due to these apparently strong genetic effects. However, the strong genetic determinism of the species-specific acoustic pattern does not prevent vocal refinement at the individual level [36]. In fact, great calls are strongly individually distinguishable in wild, adult agile gibbons, suggesting some scope for vocal modification [37].

Here, we hypothesized that the variation in gibbon mother-daughter co-singing behavior links with developmental changes in mother-daughter social bonding. So far, most studies on development of vocal behaviors in nonhuman primates have focused on the developmental change of vocalization during infancy, concluding little social influence on their acoustic structure [16]. Later potential changes at the individual level have been very rarely investigated. Moreover, very little attention has been paid to the dynamic of vocal interactions between offspring and mothers, the primary attachment figure. Despite of some evidences of routine observations of co-singing interaction between mother and daughter [34], [35], [38], functions of mother-daughter co-singing has been unclear. Therefore, it was legitimate to focus on female songs in order to investigate potential roles of mother-daughter dynamic vocal interactions in terms of social and vocal development. We observed singing interactions between mothers and sub-adult daughters, prior to emigration, in six pairs of free-ranging agile gibbons (*Hylobates agilis agilis*). To examine roles of mother-daughter co-singing, we investigated acoustic variation between daughters, measuring some behavioral variables of co-singing behaviors, i.e., co-singing rate, temporal precision of co-singing synchronizations, and mother-daughter acoustic resemblance. The co-singing rate could be analyzed as an indicator of social bond strength between mothers and daughters, because Geissman and Orgeldinger [33] have shown that duet rate is positively correlated with social grooming and spatial proximity in adult gibbons. Furthermore, recent study in songbird duetting revealed that temporal precisions of co-singing strongly correlated with pair duration [39]. Some studies reported that acoustic resemblance also correlated with social bond strength in monkeys [8], [10]–. Hence, if social factors mediate acoustic variation observed in co-singing behaviors, all the above variables should co-vary. Additionally, we examined vocal flexibility in mothers during co-singing interactions. Adult female gibbons are known to adjust their singing pattern to organize duetting with a male [41], thus we also examined vocal flexibility in mothers. We further tested a mother’s vocal adjustment ability by comparing the acoustic pattern of songs co-sung with and without the daughter. Based on the analyses, we will discuss the relevance of the two potential hypotheses, i.e. genetic versus social based individual vocal development. This is the first report examining potential roles of mother-daughter dynamic vocal interactions in nonhuman primates.

## Materials and Methods

### Ethical Note

This study was approved by Government of Indonesia and the National Institute of Sciences (LIPI), as recorded in document number 4670/SU.3/KS/2005, and complied with the Guide for the Care and Use of Laboratory Primates (Second Edition of Primate Research Institute, Kyoto University). Our observational methodology was reviewed and approved by Bogor Agricultural University prior to the study. We affirm that our observations and recordings of the gibbons did not interfere with the animals’ environment in any way. Some of the data may be available upon request.

### Subjects and Study Site

**Figure 1:**
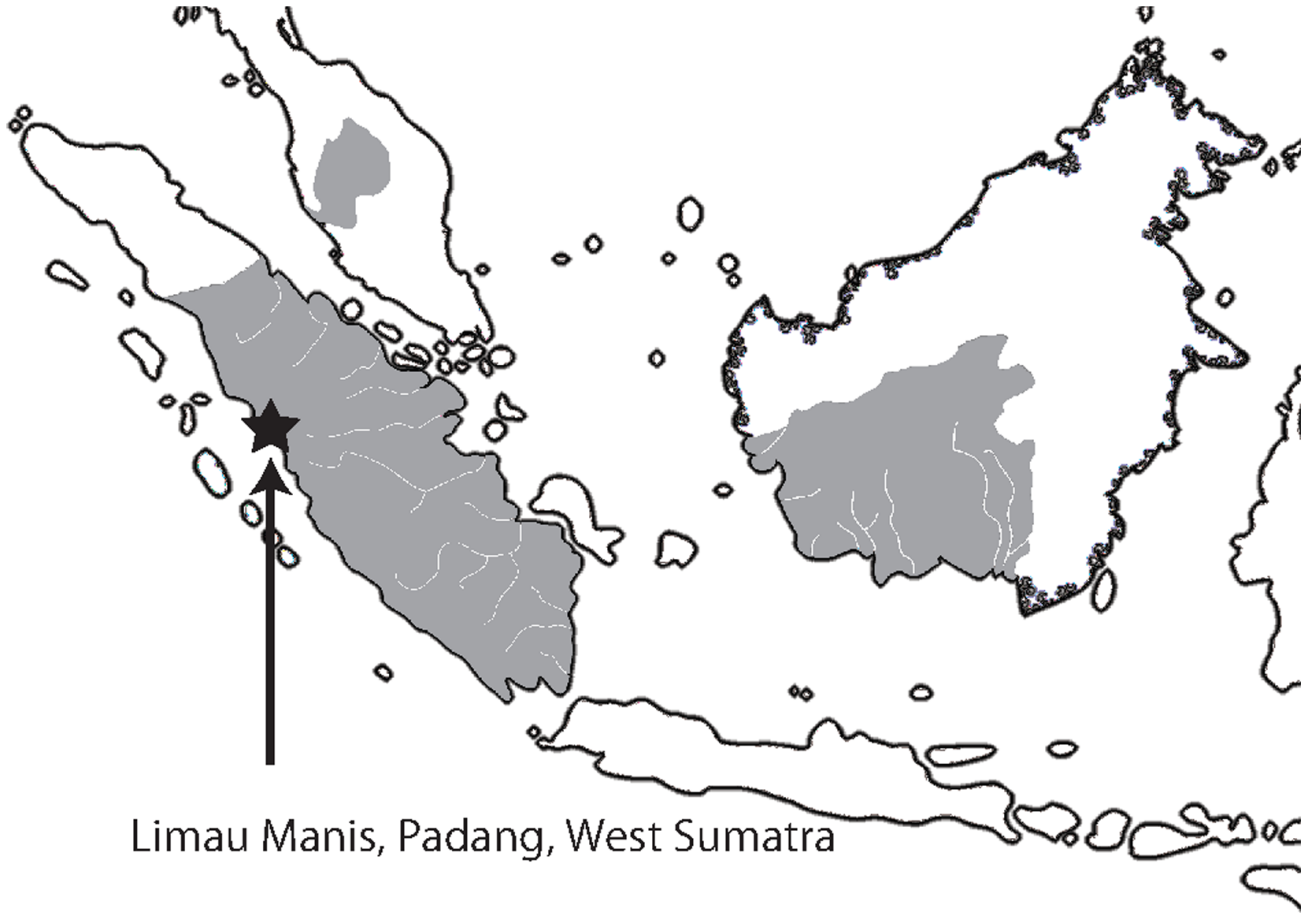
Map of Sumatra, Borneo and Malay Peninsula, illustrating the geographic range of agile gibbons (grey area). Star shows the location of our study site (0°54’S, 100°28’E, Limau Manis, Sumatra, Indonesia).

We investigated a population of free-ranging agile gibbons. Their habitat was a tropical rainforest in Limau Manis (0°54’S, 100°28’E), Sumatra, Indonesia, which consisted of a mixture of primary and secondary forest. Research took place from February and September to November 2005. Our previous investigation in this study area (Figure 1) revealed the presence of more than seven gibbon groups with known home ranges [37].

We focused on six groups. Each subject group included a mating pair and their dependent offspring (B, H, N, S, T and Z), in the vicinity of the research station. These gibbons were habituated to human researchers and had been identified during direct observations and a preliminary population census [37]. At the time of the census, each of the six groups contained one sub-adult daughter. Four groups (T, S, N, H) were also composed of one juvenile or infant (too young to confirm their sex). Based on body size definitions of closely related gibbon species in the wild [38], the ages of these daughters were estimated to be within the range of sub-adult (approximately 6 years) to fully mature adult (approximately 8 years). Based on facial fur color patterns, we classified these gibbons as female [42]. All present field observers (HK, CO, and five other colleagues) agreed with the above classification.

### Behavioral Observations and Recordings

We recorded the songs of agile gibbons between 0500 h and 1200 h via *ad libitum* sampling methods [43]. We recorded in the morning, as song rates were high at that time. Audio recording was conducted with a digital audio stereo tape recorder (Sony TCD-D100) with a sampling rate of 48 kHz and 16-bit resolution. The left audio channel was connected to a directional microphone (Sony ECM-672) and the right audio channel was connected to a lavalier microphone (Audiotechnica AT805F) for simultaneous recordings of both gibbon songs and observer comments. The callers’ identities were continuously monitored, which was relatively straightforward as gibbon sound units are long (mean ± se: 19.7±1.60 min), and gibbon families are small (two to six members). During recordings, groups were positioned not more than 50 m from observers. The total recording time was 81 h.

### Behavioral Analysis of Mother-daughter Co-singing

The mated pair duets of agile gibbons are typically divided into three consecutive sequences (the introduction, organization, and the great call) [37], [44], [45]. A duetting episode typically starts with introductory sequences, which is followed by several bouts of organizing and great call sequences, lasting several minutes in total [45].

We sampled at least six duetting episodes per group that lasted more than 10 min each, and our analysis focused on great call sequences (Table 1). In total, 46 duetting episodes were analyzed. We counted the number of occurrences of adult female great calls, as well as the number of co-singing with daughters. We identified 381 mother great calls, including 193 examples of duetting between mothers and daughters. Unlike mothers, daughters almost never sang alone; only one occurrence of a daughter singing on her own was observed (in N group).

**Table 1:** Co-singing rates of daughter and maternal preceding rates.

	Group					
	B	H	N	S	T	Z
Number of duetting episodes	9	10	6	7	6	9
Number of recorded mother’s great calls^1^	40	51	122	50	43	75
Number of recorded co-singing great calls	27	23	58	20	38	28
Co-singing rate (CR, %)	67.5	45.1	47.5	40.0	88.3	37.3
Number of co-singing great calls when the mother initiated	27	12	35	6	31	16
Number of co-singing great calls when daughter initiated	0	10	17	10	0	11
Number of non-analyzable co-singing great calls^2^	0	1	6	4	7	1
Maternal preceding rate (MPR, %)	100	54.5	67.3	37.5	100	59.3

1 Mother sung great calls with daughters (co-singing) or without daughter (solo singing). Therefore, those numbers are summations of both singing patterns. Solo great calls were used in Figure 5.

2 Because the great call is a long series of multiple calls lasting approximately 10 sec, we could readily identify the occurrence of co-singing; however, the onsets of two great calls were difficult to determine in some cases because of background noise and were considered non-analyzable.

**Figure 2:**
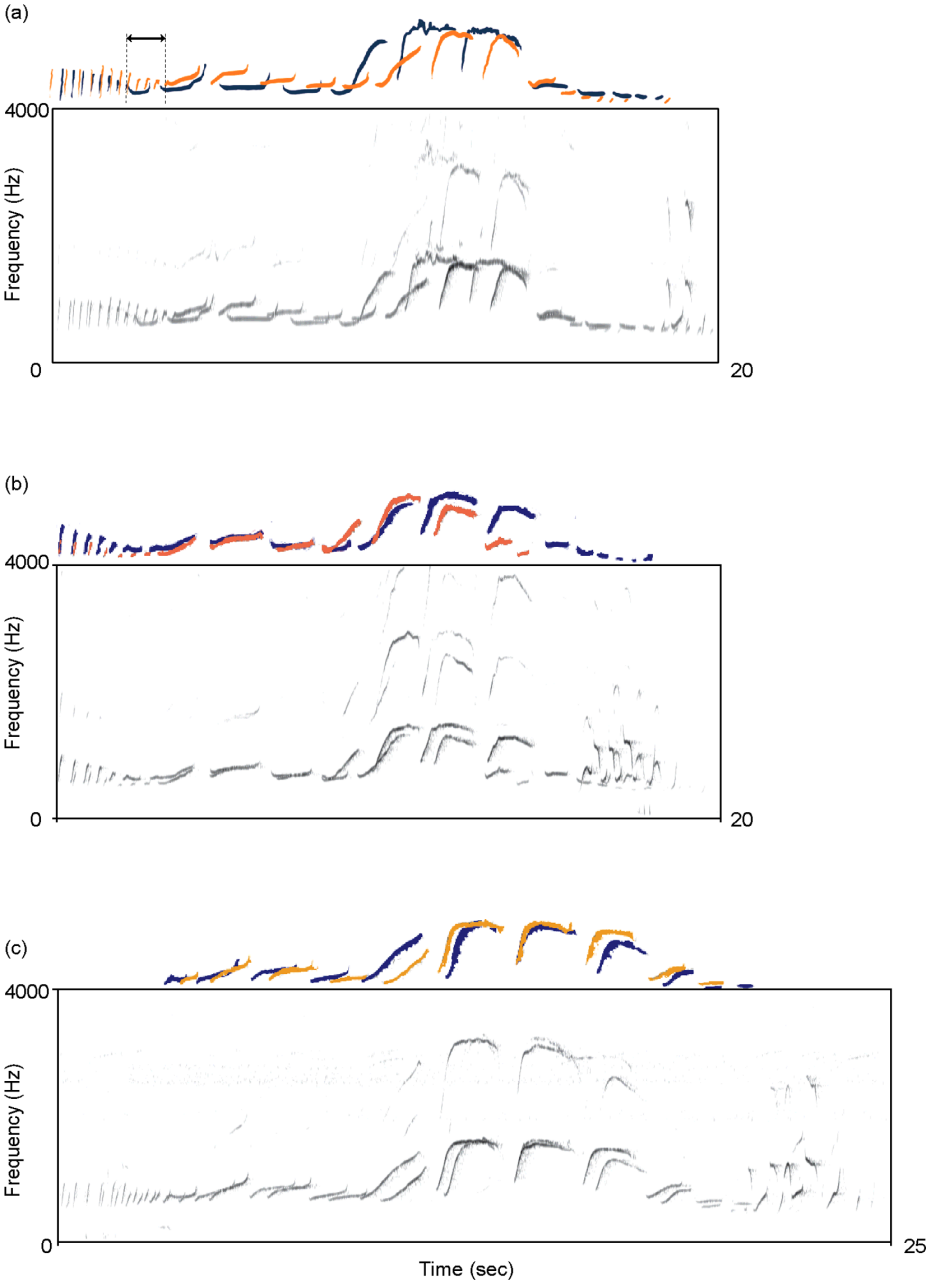
Sonograms of mother-daughter co-singing. The mother and daughter pitch contours are illustrated above the sonograms in blue and orange, respectively. The arrow represents the onset time lag between mother and daughter songs. Sound files of those examples are available in the electronic supplementary material. (Sound S1) co-singing of B group (similarity index = 0.253); (Sound S2) H group (0.398); and (Sound S3) Z group (0.551).

A typical example of a mother-daughter co-singing great call is shown in Figure 2. We calculated two behavioral rates: co-singing rates of a daughter in a mother’s great call (CR) and maternal preceding rates (MPR). The rate of daughter co-singing was the number of mother-daughter co-singing great calls divided by the total number of mother’s great calls. As one individual always initiated the duet, we calculated the maternal preceding rate (i.e., the proportion of mothers’ great calls preceding those of daughters) based on the previous definition of the onset of a great call [37].

### Acoustic Analyses

Acoustic analyses were conducted using Avisoft SASLab Pro version 5.1 (Avisoft Bioacoustics, Berlin, Germany) and customized index software from ANA [10], [11]. All spectrograms were generated using a fast Fourier transformation (size of 512 points with an overlap of 75% and a Hamming window; frequency resolution = 15 Hz; temporal resolution = 16 ms) after resampling at 8 kHz.

To evaluate the temporal features of co-singing interactions, we measured the time lag latency (TL) between the mother’s and the daughter’s calling onsets. The time lag between song onsets has been used for evaluating the temporal features of duetting [35], [46], [47]. To analyze the global acoustic patterns of great calls, we computed inter-great call acoustic similarity indices as previously used in guenons [11] (for details on the acoustic procedure, see Method S1). This approach has the advantage of enabling the computation of a single global similarity index for each pair of spectrograms compared, despite the complexity of the multi-note gibbon songs.

We calculated five different similarity index scores based on inter-individual comparisons, assessing mother-daughter similarities, and intra-individual comparisons, assessing the level of acoustic stereotypy of mothers: 1) mother-daughter similarity index at the time of co-singing (MD_co-singing_) calculated by comparing a single great call of the daughter with the overlapping one of the mother; 2) mother-daughter overall similarity index (MD_overall_) calculated by comparing all pair combinations of great calls recorded from the daughter and mother, excluding pairs of the same co-singing events; 3) non-mother-daughter similarity index (MD_nonmother_) calculated by comparing all pair combinations of great calls recorded from the daughter and non-mother adult females; 4) acoustic variability in mother great calls uttered without a daughter co-singing (M_solo_) calculated by comparing all great calls produced alone by a given mother; and 5) acoustic variability in mother great calls co-sung with a daughter (M_co-singing_) calculated by comparing all great calls produced by a given mother with her daughter.

### Statistical Analysis

To examine the potential correlations among the aforementioned variables (i.e., co-singing rates (CR), call onset time lags (TL), maternal preceding rates (MPR), mother-daughter similarity at the time of co-singing (MD_co-singing_), and similarity between non-mothers and daughters (MD_nonmother_), we calculated a generalized linear mixed model (GLMM) to estimate fitted models through the lmer method in lme4 package for R (version 2.12.1; R Developmental Core Team, 2010). This enabled statistical modeling that included a random factor with appropriate error distributions. To avoid too many multiple comparisons of all variables, co-singing rate (CR) was an explanatory fixed factor, and the group was a random factor in the present models. We used a Gaussian distribution with an identity link function for TL, MD_co-singing_, and MD_nonmother_, whereas a binomial distribution was used with a logit link function for MPR (maternal preceding case = 1, and daughter preceding case = 0, prior to model fitting). To test for potential mother-daughter acoustic matching, we computed a GLMM to compare MD_co-singing_ and MD_overall_ with the subject group as a random effect. We used a Gaussian distribution with an identity link function.

To examine the possibility that a mother acoustically modified her calling pattern when co-singing with her daughter, we additionally performed a simple regression analysis. We calculated the differences between the level of acoustic variability when the mother was duetting and when the mother was singing alone (i.e., average M_co-singing_ - average M_solo_) for each group. Differences in acoustic variability and CR were used as dependent and independent variables, respectively.

All significance levels were set at p<0.05. For GLMM analyses, log-likelihood ratio tests were performed to examine the statistical significance of fixed factors and assess whether the removal of a factor caused a significant decrease in model fit. Hence, the degrees of freedom for log-likelihood ratio tests always differed by one.

## Results

### Variability in Co-singing Rates and Maternal Preceding Rates

**Figure 3:**
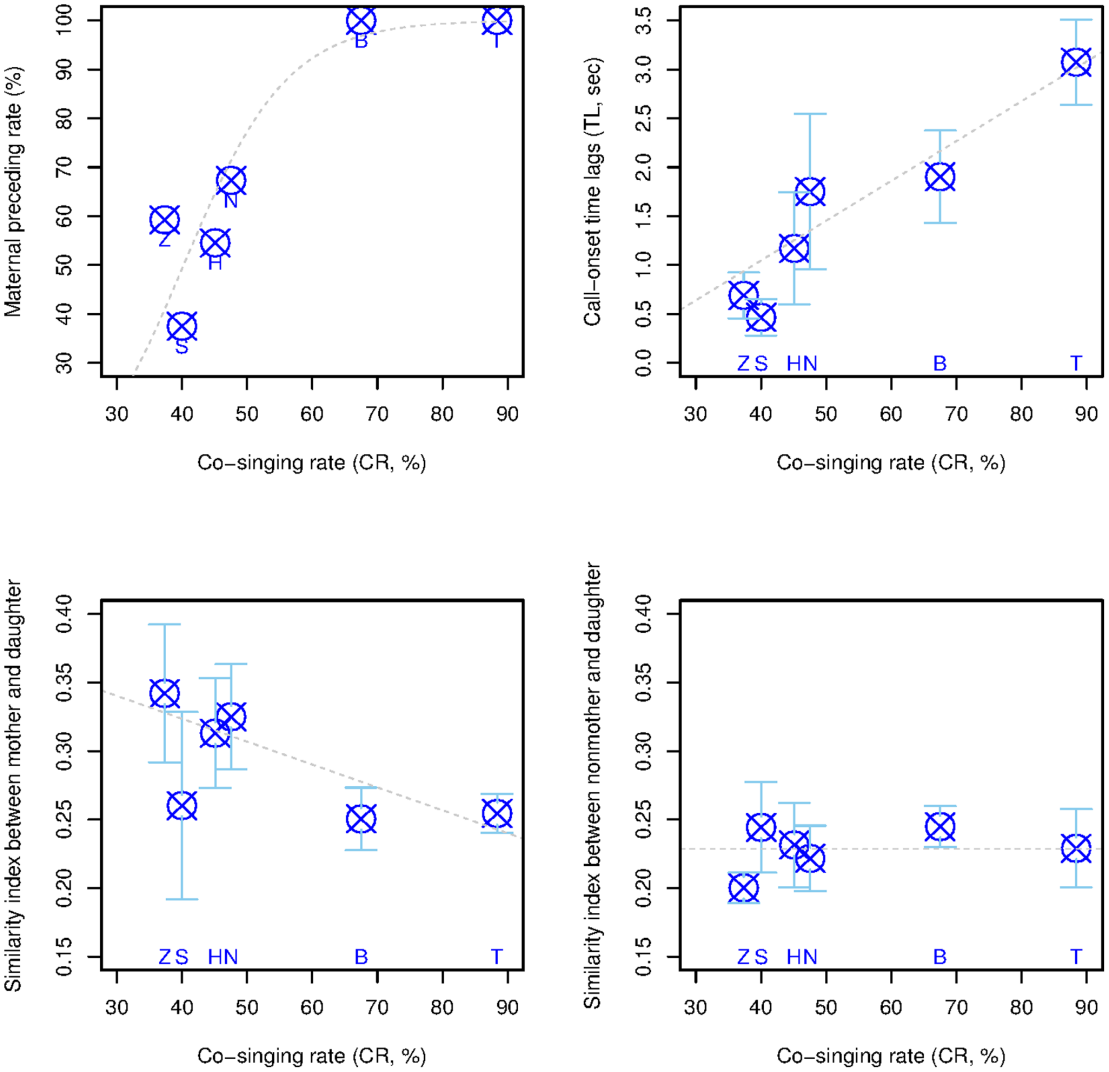
Behavioral and acoustic variables analyzed with co-singing rates. (a) Maternal preceding rates (MPRs) plotted for co-singing rates (CRs) for 6 groups. Open circles with crosses indicate the percent proportion of duet where mothers preceded the great call utterance of daughters for each group. The regression line was plotted using coefficients of estimated GLMMs using a binomial error distribution with a logit link function (for the model terms, see the statistical procedure in methods section). (b) Call onset time lags (TLs) when a mother initiated the duet plotted for co-singing rates (CRs) for 6 groups. Open circles with crosses indicate the group means, and error bars represent 95% confidence intervals. The regression line was plotted using coefficients of estimated GLMMs using a Gaussian error distribution with an identity link function. (c) Mother-daughter similarity indices at the time of co-singing (MD_co-singing_) plotted for co-singing rates (CRs) for 6 groups. Open circles with crosses indicate the group means, and error bars represent 95% confidence intervals. The regression line was plotted using coefficients of estimated GLMMs using a Gaussian error distribution with an identity link function. See Table S2 for detailed results of similarity index. (d) Similarity indices between non-mothers and daughters (MD_nonmohter_) plotted for co-singing rates (CRs) for 6 groups. Open circles with crosses indicate the group means, and error bars represent 95% confidence intervals. The regression line was plotted using coefficients of estimated GLMMs using a Gaussian error distribution with an identity link function. See Table S2 for detailed results of similarity index.

Daughters sang together with their mothers by overlapping their great calls. However, co-singing rates (CR) varied substantially among the six families, ranging from 37.3% to 88.3% of recorded mothers’ great calls. Maternal preceding rates (MPR), when mothers started the duet by singing prior to their daughters, also varied between the six groups (Table 1). The co-singing rate (CR) was positively correlated with the maternal preceding rate (MPR) (GLMM, χ^2^_1_ = 14.886, p = 0.0001, Figure 3a, Table S1), showing that mothers started duets more often when daughters co-sang more frequently.

### Temporal Controllability: Call Onset Time Lags

Co-singing rates (CR) were significantly positively correlated with mother-daughter call onset time lags (TL) when the mother initiated the co-singing (GLMM, χ^2^_1_ = 9.9903, p = 0.001, Figure 3b, Table S1), showing that daughters who decreased their co-singing rate better matched the temporal onset of their great call to that of their mothers.

### Acoustic Similarity between Mothers, Daughters, and Non-mother Adult Females

We also tested inter-group variability in the degree of mother-daughter acoustic similarity. Mother-daughter acoustic similarity at the time of co-singing (MD_co-singing_) was negatively correlated with the co-singing rate (CR) (GLMM, χ^2^_1_ = 5.251, p = 0.022, Fig. 3c, Table S1), showing that daughters who co-sang less produced great calls that were more similar to that of their mothers. We found no correlation between co-singing rate (CR) and acoustic similarity between the daughters and non-mother adult females (MD_nonmohter_) (GLMM, χ^2^_1_ = 0.0686, p = 0.79, Fig. 3d, Table S1). The similarity index between non-mothers and daughters appeared stable among groups and was not linked to co-singing rates.

### Acoustic Matching between Mother and Daughter when Co-singing

**Figure 4:**
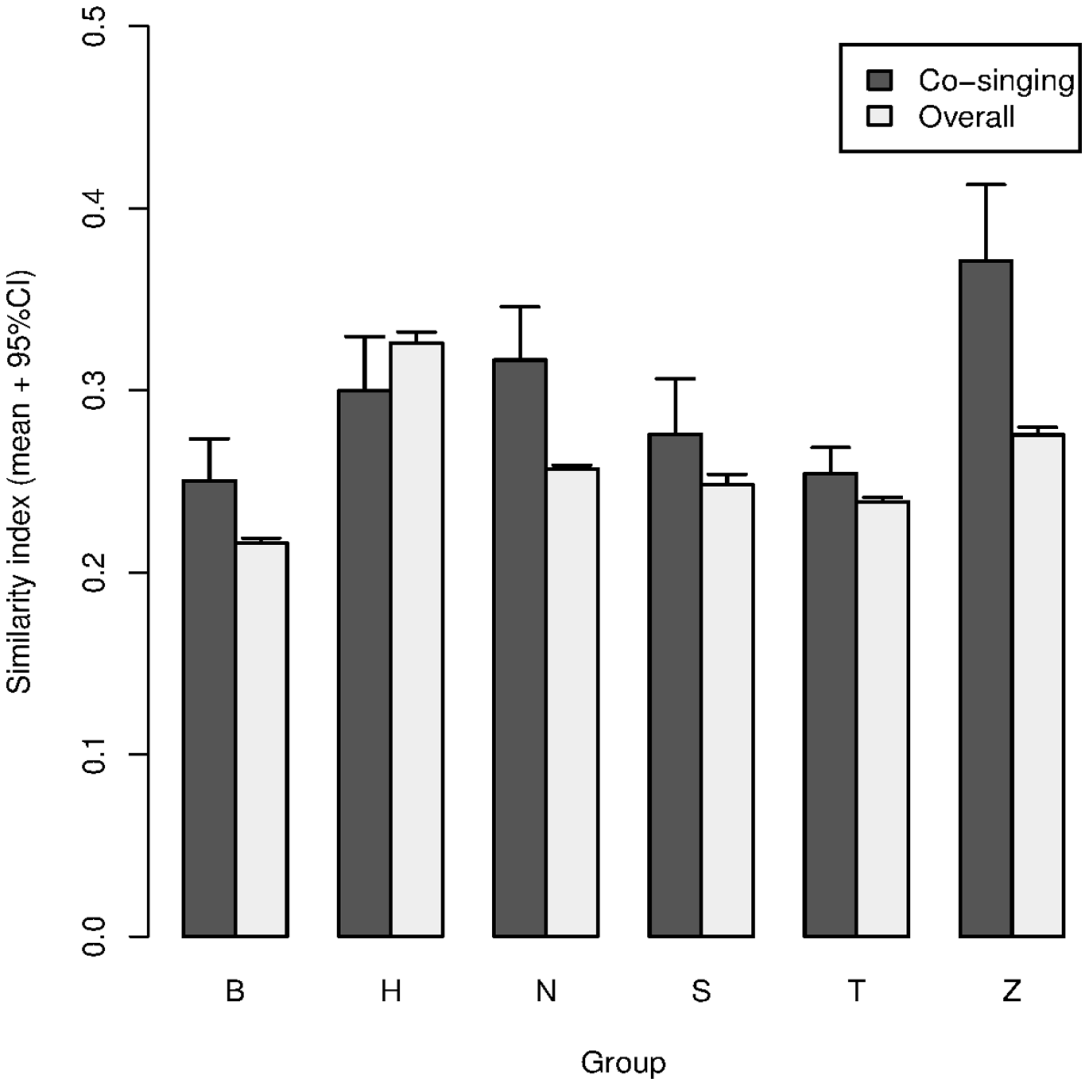
Barplot of mother-daughter similarity indices at the time of co-singing (MD_co-singing_) comparing all pair combinations of great calls recorded in the daughters and their mothers, excluding the pairs of co-singing great calls (MD_overall_), for 6 groups. Bars with error bars indicate means with 95% confidence intervals.

Mothers and daughters matched the acoustic pattern of their songs when co-singing. Mother-daughter acoustic similarities at the time of co-singing (MD_co-singing_) were significantly higher than the overall mother-daughter acoustic similarities (MD_overall_), which were calculated by comparing all possible pairs of great calls from different co-singing events (GLMM, χ^2^_1_ = 111.97, p<0.0001, Fig. 4, Table S1).

### Maternal Acoustic Adjustment when Co-singing with Daughters

**Figure 5:**
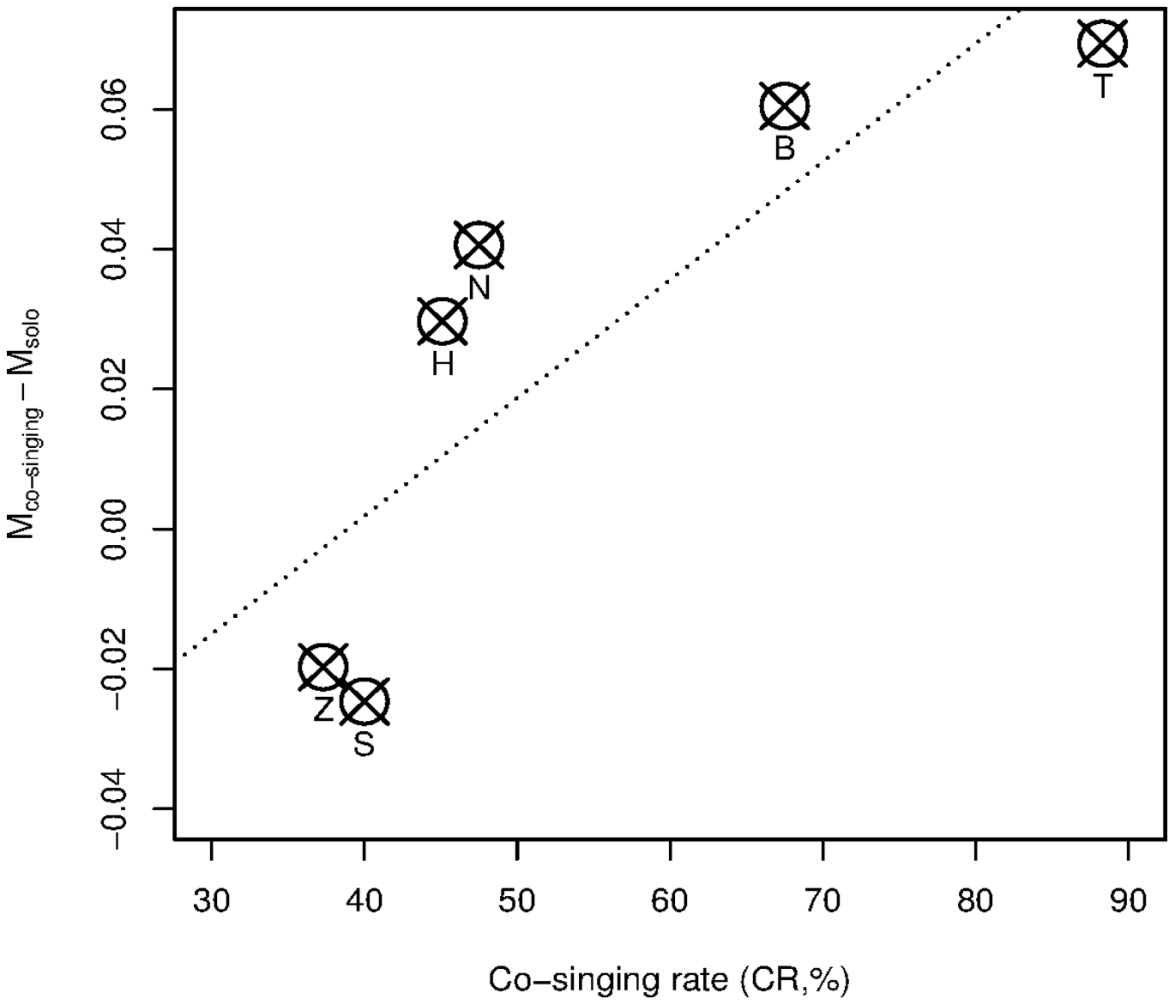
Plots of the acoustic similarity indices comparison between mother great calls uttered without any daughters (M_solo_) and in mother great calls co-sung with daughter (M_co-singing_). Here, the scores of index differences (averaged M_co-singing_ minus averaged M_solo_) are shown with co-singing rates (CRs) for 6 groups. The open circles with crosses indicate the group means. The regression line is plotted using coefficients of regression analysis. See the sample size used here (numbers of co-singing and solo great calls for each mother) in Table 1.

The mother’s vocal adjustment during co-singing with her daughter was estimated by the equation M_co-singing_ - M_solo_. This difference significantly increased in response to an increase in co-singing rates (regression analysis, *F*_1,4_ = 9.33, p = 0.038, Fig. 5). This result indicates that mothers of daughters that regularly co-sang with them were more likely to modify their own acoustic patterns, particularly when co-singing.

## Discussion

We observed significant variation in the vocal behavior and acoustic pattern of both daughters and mother gibbons during co-singing events. Co-singing rates strongly varied between the six studied groups and were negatively correlated with the temporal precision of the songs’ synchronization and with the degree of mother-daughter acoustic resemblance. In addition, we observed matching between mothers and daughters at the time of co-singing. This indicates the acoustic convergence between mothers and daughters over a short time scale, supporting the existence of context-dependent vocal flexibility in gibbons In line with this apparent case of vocal flexibility, we found that mothers adjusted the songs to a more stereotyped pattern when co-singing with their daughters than when singing alone, doing so particularly in groups with low duet rates. Altogether, these data suggest that co-singing mother-daughter duets play an important role in the vocal development of sub-adult daughters, notably concerning the development of rhythmical vocal control (improvement in temporal synchronization during duets) and acoustic characters (mother-mediated refinement of the acoustic structure of daughters’ great calls). Although more data are needed to draw more firm conclusions, our results may also highlight a potential model role played by mothers.

Demographic literature on the life history of gibbons, although still limited, has reported that co-singing rates of daughters increase progressively during the first years of life [35] but then decrease with the acquisition of social independence, from sub-adult (approximately six years old) to fully matured adult (approximately eight years old) [38]. This is the age at which a daughter usually emigrates from the natal family, forms a new pair bond, and begins duetting with her mate. The decline of co-singing has be considered an indicator of increased social independence [38] and an advanced stage of emigration (T. Geissmann and U. Reichard, personal communications). This would support the following developmental path: when acquiring social independence, daughters decrease their duet rate with mothers and become more vocally mature in terms of temporal synchronization ability and great call refined acoustic structure. However, we must acknowledge that our conclusions are based on cross-sectional data from a limited number of subjects. Currently, we can only hypothesize the trajectory of these developmental changes. Only longitudinal studies with known ages could validate this hypothesis.

To our knowledge, the current findings provide the first observational evidence of a putative maternal influence on vocal development in nonhuman primates, analogous to some features of human language acquisition. Potentially, previous studies have supported the perspective that maternal social influences do not significantly contribute to vocal development in nonhuman primates, even in gibbons. Hybrid female offspring were observed to produce calls that were acoustically distinct from both of their biological parents’ songs (white-handed gibbon, *Hylobates lar*, and pileated gibbon, *Hylobates pileatus*) [34]. Female offspring maturing in groups with genetically dissimilar parents developed acoustical patterns that were intermediate in similarity between the two species, suggesting a lack of social input [34]. However, their study of hybrid daughters measured only simple acoustic characteristics (i.e., song element number), which differs considerably between white-handed and pileated gibbons. Therefore, it seems insufficient to acoustically evaluate maternal influence at the individual (and not the species) level. Indeed, their study only showed that the overall shape of the species-specific songs was genetically determined. Our study does not refute this point. The same authors also acknowledged the possibility that daughters “practice” learning song structure via co-singing interactions [34]. Here, we focused on the detailed acoustic variation of great calls. Correlations among several cosinging-related variables suggest, to some extent, vocal-social co-development in gibbons. This would imply that vocal interactions with mothers enable vocal practice and facilitate song development through phenomenon of acoustic convergence.

Another important aspect of our study was showing that mothers modified their song structures in the presence or absence of female offspring. Mothers who co-sang more frequently initiated co-singing more often, which is in line with the idea that the mother’s song could serve as a model for the responding daughter. Given the previous findings in mother-juvenile duets where mothers were found to initiate 100% of co-singing interactions at this early stage of development [35], correlations of co-singing rates and maternal initiation rates would be mediated by developmental change of mother-daughter social relationships. More importantly, mothers who co-sang more frequently adjusted the acoustic characteristics of their songs when duetting with their daughters in order to stereotype the song. Our data confirms adult female monogamous apes’ ability regarding vocal flexibility. This is consistent with a previous finding in captive siamangs [41]. When being paired with a new male, females adjusted their song structure to the new partner identity, in parallel with the establishment of the pair bond. Here, the fact that mothers sing in a more stereotypic way with daughters is also in line with the idea that their songs can serve as a model.

Our observations, notably that daughters who duetted less with their mothers produce more mother-like vocal structures, challenge the traditional view of the fixed vocal repertoire of immature nonhuman primates. While genetic factors would determine a species’ general call structure, the local acoustic structure (e.g., call timing, frequency, and duration) would be modifiable depending on auditory feedback during vocal interactions [48], [49]. At this stage, we can only conclude in favor of a socially mediated modification of a genetically hardwired song. One hypothesis is that acoustic structures in gibbons may be modifiable by socially guided vocal learning. However, the alternative, morpho-physiological maturational (genetically driven) hypothesis, that does not require a learning process, may also explain our observation. The lack of a correlation between social independence and mother-daughter acoustic similarity when comparing the songs of the same daughters with the ones of non-mother adult females suggests that a general species-specific maturation hypothesis can be excluded. However, given that daughters likely inherit general features (e.g., body size and vocal apparatus shape) from their mothers, acoustic structures, which are partly determined by idiosyncratic morphological features, could explain some of the mother-daughter convergence during development. Future longitudinal studies, using morpho-physiological investigations and comparisons of the relative role of age and social independence, are needed to address the question of developmental mechanisms. Alternatively, it would be interesting to record captive offspring that have been accidentally deprived of a mother. We plan to examine maternal roles in vocal development within this socially isolated condition in the future.

Besides a discussion of potential maternal influences and vocal learning, our finding regarding acoustic matching during duetting is interesting for the on-going debate of vocal flexibility in nonhuman primates. Most previous findings in this area have revealed socially determined acoustic convergence on a long time scale (several months or years) [11], [12], [40], [50]. Demonstrations of a short-scale acoustic convergence (immediate) are less common [8], [13], [51]. Another limit of our study is that it is only observational. Hence, it is still unclear whether mothers and/or daughters modify to match the song characteristics of the other. This could be examined in a future study using playback experiments in which we overlap a broadcast great call stimulus when the subject (either offspring or mother) start calling. This should enable a test as to what extent mothers and daughters can modify their song structures, thus showing who is responsible for song matching.

We demonstrated the possibility that mother-daughter co-singing interactions contribute to the development of species-specific female songs in gibbons, and mothers facilitate the acquisition process in a comparable manner to humans. Our studies focusing on dynamic singing interactions not only provide evidence of vocal flexibility but also open new lines of research approaches regarding maternal contributions to vocal development in primates. Several non-exclusive arguments might explain the convergent mode of vocal developmental processes between gibbons and humans. First, gibbons are closely related to humans, suggesting shared neurological foundations. Interestingly, brain regions responsible for rhythmical motor control are more similar to those of humans than those of monkeys [52]. Second, gibbons’ arboreal life in the canopy places an emphasis on auditory communication rather than visual communication styles, as dense vegetation in the canopy of tropical rainforests restricts visual contact, particularly for small monogamous groups [29], [53]. Third, mother-offspring attachments in gibbons are stable for a relatively long time span. Given gibbons’ monogamous social systems, parents are the main social partners for daughters. The roles of parents on vocal development would be important as it is for humans [38]. Our data does not contribute to our understanding of the potential role of the father, who also exchanges songs with his mate and offspring. Given the existence of sex-specific acoustic structures, the impact of fathers on the songs of sons is another attractive avenue for future research. Additional comparative research is needed to determine whether these findings in a nonhuman primate species are precursors to certain language characteristics or simply reflect convergent evolution as a result of comparable socio-ecological constraints.

## Supporting Information

Table S1 **Result notes for the 5 GLMMs performed in the study.**
(DOC)

Table S2 **Summary of similarity index analyses.**
(DOC)

Method S1 **The detail procedure of similarity index calculation.**
(DOC)

Sound S1 **Co-singing of B group.**
(WAV)

Sound S2 **Co-singing of H group.**
(WAV)

Sound S3 **Co-singing of Z group.**
(WAV)
