# Supplementary material for: Possible Role of Mother-Daughter Vocal Interactions on the Development of Species-Specific Song in Gibbons
Source: PLoS One. 2013 Aug 12;8(8):e71432. doi: 10.1371/journal.pone.0071432 (PMC3741147; doi:10.1371/journal.pone.0071432)
Supplement: Table S1 — Result notes for the 5 GLMMs performed in the study. (DOC) [file pone.0071432.s001.doc]

**Supporting information of “Possible role of mother-daughter vocal interactions on the development of species-specific song in gibbons” by Koda et al.**

**Supporting Results**

**Supporting Table S1. Result notes for the 5 GLMMs performed in the study.**

|  | Models^#1^ | df | AIC | Log likelihood | Chi^#2^ square | df^#3^ | P^#4^ |
| --- | --- | --- | --- | --- | --- | --- | --- |
| Figure 3a | Full model | 4 | 167.25 | -80.625 | 14.886 | 1 | 0.0001 |
|  | Null model | 3 | 180.14 | -88.068 |  |  |  |
| Figure 3b | Full model | 4 | 464.91 | -228.46 | 9.99 | 1 | 0.00157 |
|  | Null model | 3 | 472.90 | -233.45 |  |  |  |
| Figure 3c | Full model | 4 | -254.53 | 131.27 | 5.251 | 1 | 0.0219 |
|  | Null model | 3 | -251.28 | 128.64 |  |  |  |
| Figure 3d | Full model | 4 | -67786 | 33897 | 0.0686 | 1 | 0.793 |
|  | Null model | 3 | -67788 | 33897 |  |  |  |
| Figure 4 | Full model | 4 | -14881 | 7444.5 | 111.97 | 1 | <0.0001 |
|  | Null model | 3 | -14771 | 7388.5 |  |  |  |

The GLMM detailed results referring to the analyses shown in Figures 3 and 4 are listed above.

^#1^Full is the model including an explanatory fixed factor with the group as a random factor, while null is the model including only the intercept as an explanatory fixed factor with the group as a random factor. ^#2^The chi-square value for log likelihood ratio tests between the full and null models. ^#3,#4^ The values for log likelihood ratio tests (shown in the main text)
