# Supplementary material for: Possible Role of Mother-Daughter Vocal Interactions on the Development of Species-Specific Song in Gibbons
Source: PLoS One. 2013 Aug 12;8(8):e71432. doi: 10.1371/journal.pone.0071432 (PMC3741147; doi:10.1371/journal.pone.0071432)
Supplement: Table S2 — Summary of similarity index analyses. (DOC) [file pone.0071432.s002.doc]

**Supporting information of “Possible role of mother-daughter vocal interactions on the development of species-specific song in gibbons” by Koda et al.**

**Supporting Results**

**Supporting Table S2. Summary of similarity index analyses.**

|  | | Group | | | | | |
| --- | --- | --- | --- | --- | --- | --- | --- |
|  | | B | H | N | S | T | Z |
| Similarity index between mother and daughter | Mean | 0.250 | 0.313 | 0.325 | 0.260 | 0.254 | 0.342 |
|  | Upper | 0.273 | 0.353 | 0.363 | 0.329 | 0.269 | 0.392 |
|  | Lower | 0.228 | 0.273 | 0.289 | 0.192 | 0.240 | 0.292 |
| Similarity index between nonmother and daughter | Mean | 0.245 | 0.231 | 0.221 | 0.244 | 0.229 | 0.200 |
|  | Upper | 0.260 | 0.262 | 0.246 | 0.277 | 0.258 | 0.211 |
|  | Lower | 0.230 | 0.200 | 0.198 | 0.211 | 0.200 | 0.190 |

The values shown above represent the means and 95 percent confidence intervals (upper-lower limits). All data are plotted in Fig 3c and Fig 3d.
