# Supplementary material for: Possible Role of Mother-Daughter Vocal Interactions on the Development of Species-Specific Song in Gibbons
Source: PLoS One. 2013 Aug 12;8(8):e71432. doi: 10.1371/journal.pone.0071432 (PMC3741147; doi:10.1371/journal.pone.0071432)
Supplement: Method S1 — The detail procedure of similarity index calculation. (DOC) [file pone.0071432.s003.doc]

**Supporting information of “Possible role of mother-daughter vocal interactions on the development of species-specific song in gibbons” by Koda et al.**

**Supporting Method S1**

**Similarity index calculation**

Given their frequency modulation pattern and sequential organisation, gibbons’ great calls are suitable for acoustic similarity index calculations, which are commonly used with whistle-like sounds of birds, cetaceans and guenons to assess the overall resemblance of complex acoustic structures [1-4]. It would be impossible to analyze the overlapping signals reliably by more traditional acoustic analysis (i.e. autocorrelation algorithms or cepstram analysis based on the Fast Fourier Transformation). Hence, we used the similarity index calculation available in ANA software [1,5]. The similarity indices were based on pixel by pixel comparisons between pairs of spectrograms. Each pixel was associated with a gray value ranging from 0 to 255. If one or both of the compared pixels had a gray value of zero, a score of “0” was given. If the two compared pixels differed by less than 16 in their gray values, a score of “2” was given. All other combinations were given a score of “1”. The total of all scores was then divided by the total number of pixels in both spectrograms with a grey value above zero. This allowed us to generate a similarity index ranging between 0 and 1; a score of 1 representing a perfect superposition. An algorithm performed the same operation for all possible superpositions by comparing spectrograms along the time axis, generating similarity indices for each temporal position. Once all temporal positions had been compared, the algorithm determined the highest similarity index for the two spectrograms compared. To standardize comparisons, we focused on the most stereotypic part of the great call (from the great call onset to the end of the great call’s climax), easily identifiable for both singers, allowing the exclusion of so-called terminal notes (a detailed definition is given in [6]).

1. Lemasson A, Hausberger M (2004) Patterns of vocal sharing and social dynamics in a captive group of Campbell's monkeys (*Cercopithecus campbelli campbelli*). Journal of Comparative Psychology 118: 347-359.

2. Farabaugh SM, Linzenbold A, Dooling RJ (1994) Vocal plasticity in budgerigars (*Melopsittacus undulatus*): Evidence for social factors in the learning of contact calls. Journal of Comparative Psychology 108: 81-92.

3. Hile AG, Striedter GF (2000) Call convergence within groups of female budgerigars (*Melopsittacus undulatus*). Ethology 106: 1105-1114.

4. Miller PJO, Bain DE (2000) Within-pod variation in the sound production of a pod of killer whales, Orcinus orca. Animal Behaviour 60: 617-628.

5. Lemasson A, Ouattara K, Petit E, Zuberbühler K (2011) Social learning of vocal structure in a nonhuman primate? BMC Evolutionary Biology 11: 362.

6. Oyakawa C, Koda H, Sugiura H (2007) Acoustic features contributing to the individuality of wild agile gibbon (*Hylobates agilis agilis*) songs. American Journal of Primatology 69: 777-790.
